# *EGFR* C797S mutation mediates resistance to third-generation inhibitors in T790M-positive non-small cell lung cancer

**DOI:** 10.1186/s13045-016-0290-1

**Published:** 2016-07-22

**Authors:** Shuhang Wang, Stella T. Tsui, Christina Liu, Yongping Song, Delong Liu

**Affiliations:** The Key Laboratory of Carcinogenesis and Translational Research (Ministry of Education), Peking University Cancer Hospital, Beijing, China; SUNY Stony Brook University, Stony Brook, NY 11794 USA; Weinberg College of Arts and Sciences, Northwestern University, Evanston, IL 60208 USA; Henan Cancer Hospital and the affiliated Cancer Hospital of Zhengzhou University, Zhengzhou, China

## Abstract

T790M mutation is the most common mechanism for resistance to first- and second-generation tyrosine kinase inhibitors (TKI) for epidermal growth factor receptor (EGFR). Several third-generation *EGFR* mutant selective TKIs are being explored to conquer this resistance. AZD9291 (osimertinib, tagrisso) has been approved for treatment of the metastatic *EGFR* T790M mutation-positive non-small cell lung cancer. Resistance to AZD9291 has been described. C797S mutation was reported to be a major mechanism for resistance to T790M-targeting EGFR inhibitors. This review summarizes the latest development in identifying the C797S mutation and EAI045, the novel selective inhibitor overcoming the C797S mutant.

## Background

**Fig. 1:**
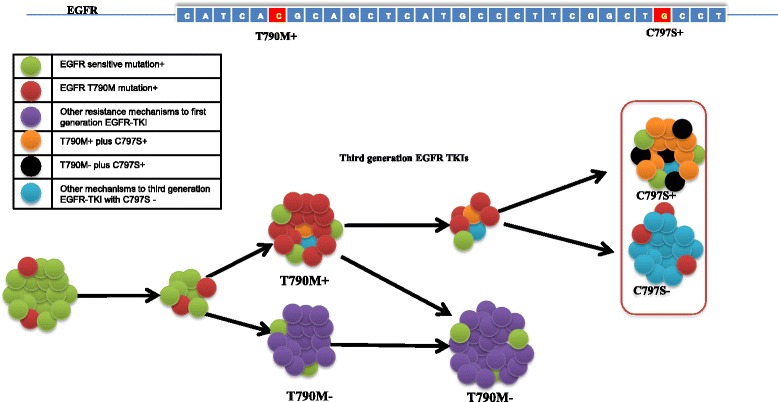
Clonal evolution of NSCLC cancer cells and mechanisms of resistance to third-generation EGFR tyrosine kinase inhibitors. The T790M and C797S mutations were highlighted in the EGFR sequence. Each colored ball represents a distinct clone. The number of balls in each group indicates relative clonal size. *NSCLC* non-small cell lung cancer, *EGFR* epidermal growth factor receptor

T790M mutation is the most common mechanism of resistance to the first- and second-generation of epidermal growth factor receptor (*EGFR*) tyrosine kinase inhibitors (TKI) [1]. Clinical trials are being done for several T790M-targeting third-generation EGFR-TKIs [2, 3]. These inhibitors include AZD9291 (osimertinib, mereletinib, tagrisso), rociletinib (CO-1686), HM61713 (BI 1482694), ASP8273, EGF816, and PF-06747775 [4–10]. AZD9291 has been shown to have a response rate (RR) of 61 % in *EGFR* T790M-positive non-small cell lung cancer (NSCLC) patients [4, 10]. HM61713 at 800 mg/day showed a 58.8 % response rate [5]. Unfortunately, these lung cancer patients eventually developed resistance to these drugs after 10 months. A better understanding of the mechanisms of resistance to these third-generation EGFR inhibitors is critical for developing new strategies to treat these patients [11]. *EGFR Cys797Ser* (C797S) mutation, located within the tyrosine kinase domain, was recently reported to be a potential mechanism of resistance to irreversible *EGFR* inhibitors such as AZD9291, HM61713, WZ4002, and CO-1686 in T790M-positive patients [12–16] (Fig. 1). This article reviewed the latest development in identifying the C797S mutation and other mechanisms of resistance.

## C797S mediates resistance to AZD9291

In the first-in-human phase I/II AURA trial of AZD9291, systemic progression in NSCLC patients was seen after treatment for a median of 9.6 months [10]. Characterization of the mechanisms of resistance in 22 patients who became resistant to AZD9291 was reported [12]. These patients with progression on AZD9291 in the AURA trial had paired pre-treatment and post-treatment plasma samples. Cell-free DNA (cfDNA) from the plasma of these patients was analyzed by next-gene sequencing (NGS). All EGFR coding exons were analyzed through a 20-gene panel. In the index case, an acquired T → A mutation encoding an *EGFR* C797S mutation was identified. In another case, an acquired C797S from G → C mutation was documented. This group established a Ba/F3 cell line harboring the C797S mutation and confirmed that the cell line was resistant to AZD9291. Through the study of T790M-positive patients with acquired resistance to AZD9291, three molecular subtypes of AZD9291 resistance were revealed: T790M*+/*C797S+, T790M*+/*C797S−, and T790M*−/*C797S−. The report also discovered that in some cases, two different nucleotide mutations (T to A and G to C) leading to C797S amino acid mutation occurred in the same patients. Since only 6 out of 15 cases acquired C797S mutation, additional mechanisms of resistance to AZD9291 must be present.

In a separate case report, a female non-smoker with widely metastatic lung adenocarcinoma progressed through first-line chemotherapy and second-line erlotinib [17]. She was found to have the *EGFR* 19 deletion (del 19) and T790M at this point. She was enrolled in the phase 1 AURA study of AZD9291 (NCT01802632) and received AZD9291 for 9 months prior to disease progression. Tumor biopsy at this juncture showed the *EGFR* C797S mutation, in addition to the del 19 and T790M. Under the strong selective pressure of EGFR-TKIs, the tumor developed secondary T790M and tertiary C797S mutations in the *EGFR* gene to bypass the TKIs and maintain EGFR signaling.

## C797S mutation mediates resistance to HM61713

HM61713 (BI 1482694) is another third-generation EGFR inhibitor and covalently binds to a cysteine residue near the kinase domain of mutant EGFR [18, 19]. In a phase I/II study, HM61713 was shown to be active for patients with T790M-positive NSCLC [5].

The first case report on resistance to HM61713 was on a 57-year-old female never-smoker with stage IV lung adenocarcinoma harboring *EGFR* del 19 [13]. The patient developed T790M mutation and became refractory to gefitinib. She was enrolled into the trial of HM61713 and was progression free for 17 months. After progression, a repeat biopsy was performed and C797S mutation was found in addition to T790M mutation and del 19. Therefore, the tertiary acquired C797S mutation conferred resistance to another third-generation EGFR TKI.

## Exploration of mutations mediating resistance to third-generation TKIs

To search for acquired resistance mutations in *EGFR* gene, a group from Dana Farber Cancer Center utilized site-directed mutagenesis in *EGFR* mutant Ba/F3 cell lines harboring sensitizing mutations and/or T790M [14]. The cells were then treated with third-generation TKIs, WZ4002, CO-1686, and AZD9291. Resistant clones were selected out, and mutations were characterized. Three major resistant mutants were identified as *EGFR* L718Q, L844V, and C797S*.* All of the three mutations could cause resistance to both WZ4002 and CO-1686. Only C797S mutation confers AZD9291 resistance. Most interestingly, in the presence of del 19 or L858R and T790M, C797S mutation leads to resistance to all current *EGFR* inhibitors (gefitinib, afatinib, WZ4002, CO-1686, and AZD9291), but L858R/T790M/C797S mutant remains partially sensitive to cetuximab. It remains to be determined whether cetuximab or cetuximab-based combinations are effective clinically in NSCLC patients that develop the L858R/T790M/C797S mutant clone.

In a separate study, a cell line, MGH121, was established from pleural effusion of a NSCLC patient who became resistant to erlotinib [15]. This cell line was sensitive to the third-generation TKIs, including WZ4002, CO-1686, and AZD9291. MGH121 cells were treated with increasing doses of a third-generation TKI, WZ4002. This led to MGH121 Res#1 which was resistant to third-generation TKIs. C797S was found to be the acquired mutation. When the L858R/T790M/C797S mutant construct was stably expressed in MGH121, the cells became resistant to all EGFR TKIs. The study explored further effect of the presence of T790M and C797S together in the same allele (i.e., *cis*) or in a different allele in the same cell (i.e., *trans*) on the sensitivity to TKIs. It was clearly demonstrated in the in vitro system that del19/T790M was resistant to the second-generation TKIs, whereas del19/C797S was resistant to the third-generation inhibitors. When T790M and C797S were present in *cis*, the cells were resistant to all EGFR TKIs. Therefore, characterization of mutation status may guide clinical decision on therapeutic approaches.

## *HER2* and *MET* amplification mediates resistance to AZD9291

Since some patients who progressed on AZD9291 were negative for the C797S mutation, additional resistance mechanisms must be present. In a case report, a 54-year-old male with stage IV adenocarcinoma was found to have acquired T790M mutation after progression from second-line treatment with gefitinib [20]. The disease progressed after 12 months of AZD9291 treatment on the AURA trial. *HER2* amplification was identified without C797S mutation from the tumor biopsy.

The second patient from the same report was a 60-year-old female, a never-smoker, who was diagnosed with stage IV adenocarcinoma with pleural metastasis [20]. Mutation analysis revealed the known *EGFR* activating mutation in exon 21, L858R. She received erlotinib and gefitinib sequentially for 12 months. After disease progression, T790M was identified by NGS and she was treated with AZD9291 on the AURA trial [10]. She had partial tumor regression and remained progression free for 10 months. Re-biopsy of the AZD9291 resistant tumor identified an *EGFR* activating mutation and *CMET* amplification without T790M or C797S mutation. These two cases indicated that in refractory NSCLC without T790M or C797S mutations, additional gene mutations or amplifications of tyrosine kinases other than EGFR can be the mechanisms of resistance. Additional treatment targeting the mutations will be needed.

## EAI045, a fourth-generation selective inhibitor overcoming EGFR C797S

Using purified EGFR mutant kinase peptide containing L858R/T790M mutations, a library of approximately 2.5 million compounds were screened to search for selective inhibitors against the kinase mutant [21]. EGFR allosteric inhibitor-1 (EAI001) was found to have such selectivity toward the EGFR mutant. Further optimization of this compound through medicinal chemistry yielded a highly selective inhibitor, EAI045, toward L858R/T790M mutant (IC50 = 3 nM). EAI045 was found to have a 1000-fold selectivity for the mutant versus wild-type EGFR. The compound is an allosteric inhibitor, rather than an ATP-competing agent. EAI045 was confirmed to be highly selective against a panel of 250 protein kinase peptides. However, EAI045 was not able to completely abolish EGFR autophosphorylation in H1975 NSCLC cell line harboring the L858R/T790M mutant. Since EGFR dimerization is required for kinase enzyme activation [22–24], the investigators hypothesized that EAI045 was active against one subunit of an EGFR heterodimer/asymmetric dimer [21]. It was confirmed that dimerization-defective/independent mutants were markedly more sensitive to EAI045. When combined with cetuximab that blocks EGFR dimerization [25], EAI045 markedly reduced tumor growth in a mouse model of L858R/T790M—mutant-driven lung cancer. The mice treated alone with EAI045 did not respond. EAI045 in combination with cetuximab also induced marked tumor shrinkage in the mouse model carrying L858R/T790M/C797S, a mutant known to be resistant to all third-generation EGFR TKIs. EAI045 and cetuximab exhibited mechanistic synergy. EAI045 represents a novel selective inhibitor that can overcome T790M and C797S resistance mutations [21].

## Discussion

C797S mutation in the *EGFR* gene was found to confer resistance clinically to third-generation TKIs, AZD9291 and HM61713. This likely represents a tertiary acquired mutation that mediates resistance to all known third-generation EGFR TKIs. Some cases were found to harbor two independent clones of C797S mutation, while others even became T790M negative, indicating the heterogeneity of malignant cells. This remains a significant challenge for treatment of lung cancers.

C481S mutation has been reported to mediate resistance to ibrutinib, the first-in-class irreversible Bruton tyrosine kinase (BTK) inhibitor [26–29]. Ibrutinib covalently binds to the cysteine residue 481 in the BTK. This may suggest that the change from cysteine residue to serine may be a recurring mutation that can block inhibitor binding to a broad range of tyrosine kinases. Targeted sequencing for cysteine residue codon mutations may represent a new method to rapidly identify mutations in other tyrosine kinases that harbor similar cysteine-containing motif in the tyrosine kinase domain.

Since more and more TKIs targeting EGFR and anaplastic lymphoma kinase (ALK) mutations are being used for NSCLC therapy [30–34], molecular testing guidance has been established [35]. The guideline suggests that lung cancer tissues are tested by PCR for EGFR mutations and by FISH for ALK mutations [35]. Liquid biopsy is increasingly used for cancer diagnosis and therapy monitoring [36]. cfDNA was used for screening of EGFR mutations. This led to the discovery of C797S mutation [12]. This technology of testing cfDNA is becoming an important companion tool for biomarker analysis and facilitating drug development [37–41].

With the availability of AZD9291 for clinical treatment of T790M-positive NSCLC, more and more resistant cases will appear. Additional mechanisms of resistance to third-generation TKIs, such as *HER2* and *MET* amplification in C797S negative cases, were identified. Loss of T790M mutation was another mechanism of resistance to AZD9291 [12]. Due to the vast diversity of malignant clones, combination therapy of AZD9291 with other agents will be needed to overcome the spectrum of resistant clones [42]. AZD9291 in combination with MET inhibitors or MEK inhibitors is being explored (NCT02143466). Immune checkpoint blockers have been shown to be active in a broad range of malignancies [43–51]. Combination of AZD9291 and PD-L1 antibody is underway in the multi-arm phase Ib study (NCT02143466). Finally, through purposefully targeting allosteric sites in the EGFR tyrosine kinase domain and screening a vast library of compounds that selectively target the resistant EGFR mutant, a highly selective inhibitor, EAI045, has been discovered.

## Conclusions

*EGFR* C797S mutation mediates resistance to third-generation TKIs, AZD9291 and HM61713. Additional mechanisms of resistance are being identified. Combination therapy of AZD9291 with other agents may be one way to overcome the acquired mutation. EAI045 represents a purposefully designed selective inhibitor overcoming EGFR C797S mutation.

## Abbreviations

ALK, anaplastic lymphoma kinase; BTK, Bruton tyrosine kinase; cfDNA, cell-free DNA; EGFR, epidermal growth factor receptor; NGS, next-gene sequencing; NSCLC, non-small cell lung cancer
